# Scoring the tumor-stroma ratio in colon cancer: procedure and recommendations

**DOI:** 10.1007/s00428-018-2408-z

**Published:** 2018-07-20

**Authors:** G. W. van Pelt, S. Kjær-Frifeldt, J. H. J. M. van Krieken, R. Al Dieri, H. Morreau, R. A. E. M. Tollenaar, F. B. Sørensen, W. E. Mesker

**Affiliations:** 1 Department of Surgery, Leiden University Medical Center, Albinusdreef 2, 2333 ZA Leiden, Netherlands; 2 Department of Clinical Pathology, Vejle Hospital, part of Lillebaelt Hospital, Vejle, Denmark; 3 Department of Pathology, Radboud University Medical Center, Nijmegen, Netherlands; 4 European Society of Pathology, Brussels, Belgium; 5 Department of Pathology, Leiden University Medical Center, Leiden, Netherlands; 6 Institute of Regional Health Research, University of Southern Denmark, Odense, Denmark; 7 University Institute of Pathology, Aarhus University Hospital, Aarhus, Denmark

**Keywords:** Colon cancer, Protocol, Recommendations, Scoring, Tumor-stroma ratio

## Abstract

The tumor-stroma ratio (TSR) has been reported as a strong, independent prognostic parameter in colon cancer as well as in other epithelial cancer types, and may be implemented to routine pathology diagnostics. The TSR is an easy technique, based on routine hematoxylin and eosin stained histological sections, estimating the amount of stroma present in the primary tumor. It links tumors with high stromal content to poor prognosis. The analysis time is less than 2 min with a low inter-observer variation. Scoring of the TSR has been validated in a number of independent international studies. In this manuscript, we provide a detailed technical description of estimating the TSR in colon cancer, including examples, pitfalls, and recommendations.

**Electronic supplementary material:**

The online version of this article (10.1007/s00428-018-2408-z) contains supplementary material, which is available to authorized users.

## Introduction

For many years, the choice of optimal treatment of cancer has mostly been based on clinicopathological characteristics, such as patient age and performance status, tumor type, malignancy grade, tumor size, and the presence of regional or distant metastases [1]. Current research in biomarker development is focusing more and more on the tumor microenvironment. Molecular biomarkers based on tumor characteristics have been developed, but one should not ignore valuable information provided by the tumor microenvironment, i.e., the stromal compartment of the tumor. Tumor-stroma plays an important role in cancer initiation and progression, in that the stroma interacts with nonmalignant cells as well as with malignant cells at different stages of tumorigenesis, ranging from tumor onset to invasion and metastasis [2].

As shown by our research group, the morphological evaluation of the tumor microenvironment in conventional, routine hematoxylin and eosin (H&E) stained tissue sections provides valuable information with high prognostic impact. Epithelial malignant tumors from patients with unfavorable prognosis have been documented to show a high proportion of stroma (> 50% stroma = stroma-high), whereas tumors with abundant carcinoma tissue (≤ 50% stroma = stroma-low) are associated with a better prognosis. This phenomenon has led to the development of the tumor-stroma ratio (TSR) as a prognostic parameter. Evaluation of this parameter in large patient series has confirmed its prognostic value for several types of cancers including colon [3–6], breast [7–9] and esophageal carcinomas [10]. International groups have validated our results for colon and breast cancer, and additionally, found the same prognostic value in other types of epithelial cancer, e.g., cervical and lung cancer [11–21]. The TSR scoring technique has been shown to be highly reproducible, with inter-observer kappa-values ranging from 0.68 to 0.97 (Table 1). Owing its simplicity and reliability, the TSR may add significant prognostic information to the currently used TNM classification, and is well-suited and cost-effective for implementation in routine diagnostics by the pathologist.

**Table 1:** An overview of tumor-stroma ratio studies reporting an inter-observer score

Study	Number of patients	Stage	Type of cancer	Inter-observer variation^a^
Mesker et al., 2009 [5]	135	I–II	Colon cancer	*Κ* = 0.6–0.7^b^ (3 observers)
Courrech Staal et al., 2010 [10]	93	I–IV	Esophageal cancer	*Κ* = 0.84^b^
West et al., 2010 [17]	145	I–IV	Colorectal cancer	*Κ* = 0.97
De Kruijf et al., 2011 [7]	574	I–III	Breast cancer	*Κ* = 0.85^b^
Moorman et al., 2012 [13]	124	I–III	Breast cancer	*Κ* = 0.74^b^
Wang et al., 2012 [15]	95	I–III	Esophageal squamous cell cancer	*Κ* = 0.84^b^
Huijbers et al., 2013 [3]	710	II–III	Colon cancer	*Κ* = 0.89^b^
Dekker et al., 2013 [8]	403	I–II	Breast cancer	*Κ* = 0.80^b^
Downey et al., 2014 [22]	180	I–III	Breast cancer (ER+)	*Κ* = 0.70
Park et al., 2014 [14]	250	I–III	Colorectal cancer	*Κ* = 0.81^b^
Liu et al., 2014 [11]	184	I–II	Cervical cancer	*Κ* = 0.81^b^
Zhang et al., 2014 [19]	93	I–IV	Nasopharyngeal cancer	*Κ* = 0.85^b^
Gujam et al., 2014 [21]	361	I–III	Breast cancer	*Κ* = 0.83^b^
Lv et al., 2015 [12]	300	I–IV	Hepatocellular cancer	*Κ* = 0.87^b^
Pongsuvareeyakul et al., 2015 [23]	131	I–II	Cervical	*Κ* = 0.78^b^
van Pelt et al., 2016 [6]	102	III	Colon cancer	*Κ* = 0.73^b^
Li et al., 2017 [24]	51	II–IV	Gallbladder	*Κ* = 0.85^b^
Roeke et al., 2017 [9]	737	I–III	Breast cancer	*Κ* = 0.68^b^

^a^Kappa value

^b^Study in which the method described in this paper was used for scoring the TSR

In this paper, we describe in detail the technical protocol of determining the TSR in colon cancer, including examples, pitfalls, and recommendations.

## Method

### Slide selection

Slides of the primary tumor are selected from the most invasive part of the colon adenocarcinoma (i.e., the slides used in routine pathology to determine the T status). For retrospective studies, these slides are mostly indicated in the pathology report, and if not, all available tumor slides are collected and analyzed. In case of more slides to be analyzed from the most invasive part of the tumor, the section with the highest percentage of stroma is scored and decisive for the final estimation of the TSR.

### Histopathological scoring

**Fig. 1:**
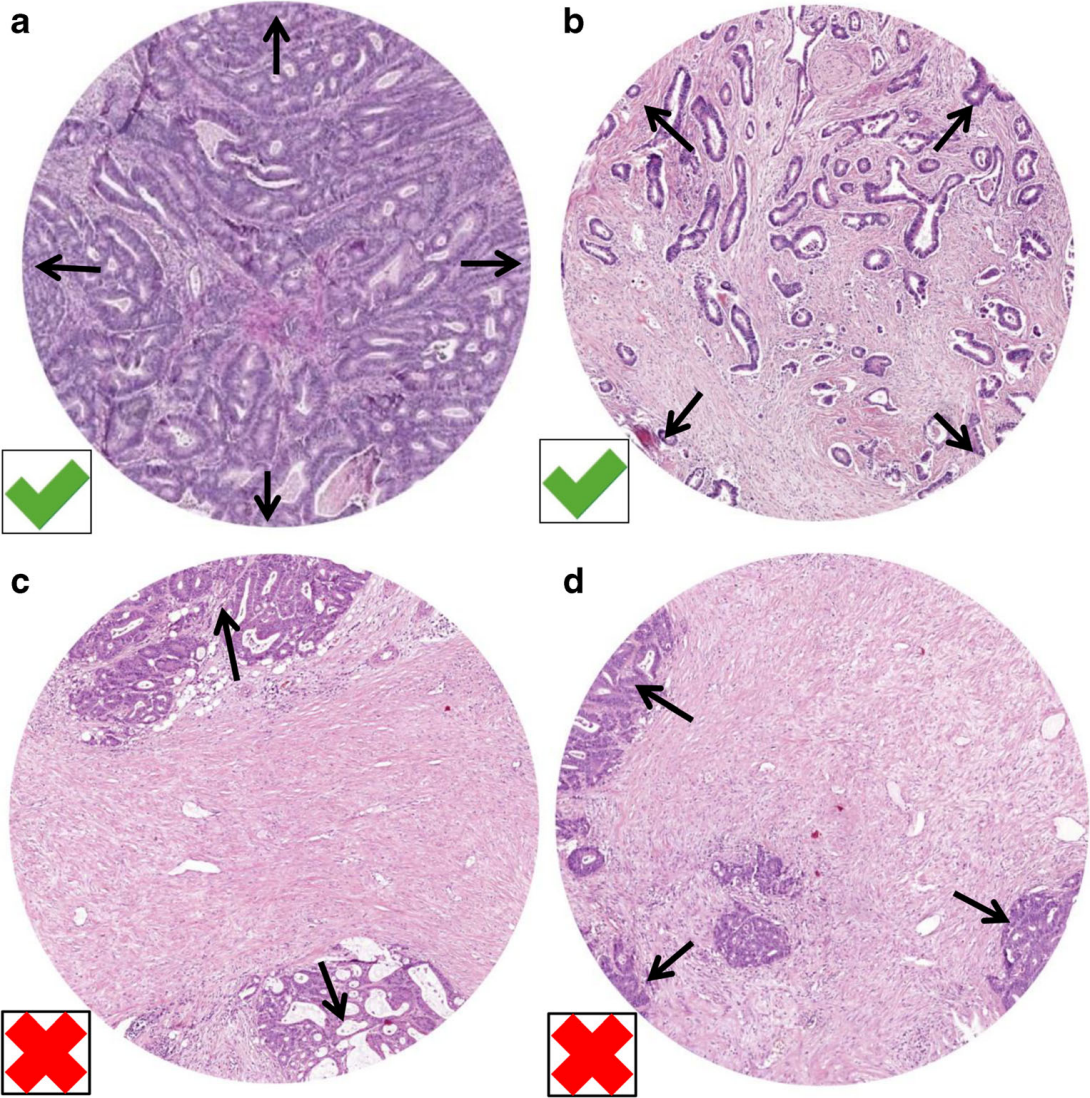
Examples of a stroma-low (**a**) and stroma-high (**b**) colon carcinoma, which meet the criteria for the presence of vital tumor cells on all four sides of the field of vision (arrows) and are thus correct for scoring. When tumor cells are only present at two (**c**) or three (**d**) sides of the field of vision (mucus is not included in estimating TSR), these areas are not suitable for scoring (Images displaying the microscopic view, all images × 100 magnification)

H&E stained tissue sections from the primary tumor of 4 μm thickness are analyzed by conventional microscopy. Areas appearing to have the highest amount of stroma are selected using the × 2.5 or the × 5 lens. Hereafter, an area where both tumor and stromal tissue are present within this vision-site is selected using a × 10 objective. Tumor cells are to be present at all borders of the selected image field (Fig. 1). The amount of stroma tissue is estimated per 10% increment (10, 20, 30%, etc.) per image field. For statistical analysis, stromal ratio groups are divided in stroma-high and stroma-low groups. Stroma-high is defined as > 50% stromal area, and stroma-low as ≤ 50% stromal area in the histological section, as determined a priori to have maximum discriminative power [4]. Even if there is only one image field with a stroma-high score, this image field is decisive.

When scoring the TSR, misinterpretations while estimating the percentage of stroma can occur due to general issues, as well as based on specific histological issues. Both are discussed below.

## General issues

### Different oculars

In daily practice, different microscopes are available, with different lens specifications, leading to different area sizes of the field of vision. With most used oculars having a diameter ranging from 18 to 22 mm, the area of the field of vision will range from 2.54 to 3.80 mm^2^. However, in exceptional cases, a larger field of vision will make it able to meet the criterion of tumor cells needing to be present at all borders, whereas with a smaller field of vision this might not be possible, or vice versa. For scoring the TSR, this has not lead to any major differences in scoring percentages.

### Quality of H&E staining

An important factor for determining the TSR is the quality of the H&E stain. When the stain is too pale or too intense, it is difficult to distinguish the stromal tissue from the smooth muscle tissue of the bowel wall. This may happen, when using too thin or too thick histologic sections, respectively.

If the TSR scoring cannot be carried out optimally due to the quality of the stain, it is recommended to re-stain the section before scoring the TSR.

### Only one possibly stroma-high area (stromal component > 50%)

In case there is only one area/field of vision that might be categorized as stroma-high, but doubt remains (even after consulting a second observer), we recommend to consider the total composition of the whole tissue section with the × 2.5 or × 5 objective to classify that particular case. However, if there is no doubt that the one and only field is stroma-high (or consensus can be reached), the case is classified as stroma-high.

## Histological issues

It is always preferred to score a field of vision in which no muscle tissue, necrotic tissue, and/or large blood vessels are present, but as this might not always be the case, we discuss the options below and provide our recommendations, also regarding other tissue qualities (see Table 2 for a summary).

**Table 2:** Summary of the difficulties occurring during scoring the tumor-stroma ratio in colon adenocarcinomas with recommendations on how to act on them

Difficulty	Recommendation
Mucinous tumor	Mucus should be ignored for scoring^a^
(Abundant) inflammatory cell infiltration	Infiltration with inflammatory cells is not an exclusion criteria and can be included in the scoring.
Necrotic tissue	Necrotic tissue should be left out of the microscopic field. If this is not possible, the necrotic parts will have to be ignored for scoring^a^
Smooth muscle tissue	Smooth muscle tissue should not be considered for scoring. In case it is not possible to select a suitable field without smooth muscle tissue (e.g., in stage II tumors), this tissue compartment should be ignored for scoring.^a^ A desmin stain may be of assistance.
Glandular lumen	Areas of glandular lumens are ignored for scoring^a^
Blood vessels	Small vessels are included as part of the stroma. Large vessels with a muscular wall (> 3 layers of smooth muscle cells) should be avoided or else ignored for scoring^a^
Tumor budding cells	Budding adenocarcinoma cells should be separated from the surrounding stroma, and may be highlighted by a cytokeratin stain (AE1/AE3 is recommended) in problematic cases.
Hyalinization	Part of the stroma and therefore included for scoring

^a^To ignore areas for scoring: the microscopic field minus the tissue that has to be visually ignored is set at 100%. The stroma percentage has to be determined from only the solid (= neoplastic + vital stromal compartment) tissue parts

### Mucinous adenocarcinomas

**Fig. 2:**
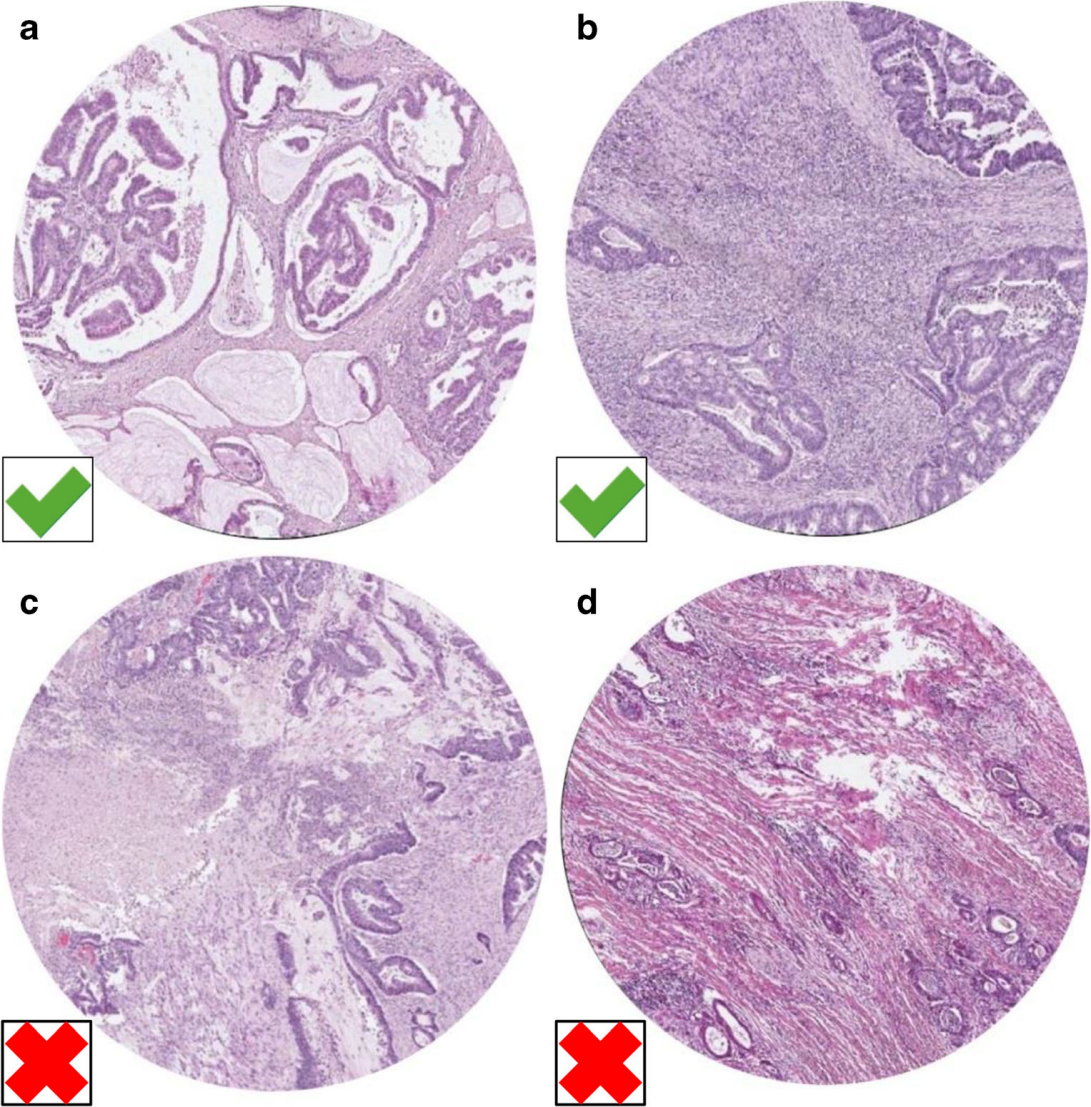
Examples of infiltration of a mucinous colon carcinoma (**a**) and inflammatory cells (**b**), which both meet the criteria for scoring. For the mucinous colon carcinoma, the mucus has to be ignored for scoring. Fields of vision with necrotic tissue (**c**) and smooth muscle tissue (**d**) do not meet the scoring criteria and should not be considered for scoring (Images displaying the microscopic view, all images × 100 magnification)

In mucinous cancers, it can be very difficult to estimate the TSR correctly. The mucus is allowed to be present in the field of vision, but has to be visually ignored from scoring (Table 2, Fig. 2a, Supplementary fig. 1). It may also be possible to determine the TSR in the non-mucinous area of a mucinous tumor’s deepest penetration of the bowel wall.

### Infiltration with inflammatory cells

Heavy inflammation is often encountered within the stromal component in the tumor microenvironment of colon adenocarcinomas, and can be included in the TSR scoring as part of the stroma. However, lymphoid follicles may represent an integrated part of the “native” histology of the large bowel, and thus may not constitute a response to the expanding epithelial tumor within the tumor microenvironment. Thus, we recommend areas with lymphocytic follicles/aggregates to be avoided or else visually ignored from scoring (Fig. 2b).

### Necrotic tissue

Necrotic tissue or areas with pure neutrophilic inflammation, which may indicate necrosis, should be left out of the microscopic scoring field. If this is not possible, the necrotic parts will have to be visually ignored for scoring, as for the mucus in mucinous tumors (Table 2, Fig. 2c).

### Lumen

Almost all tissue sections from colon adenocarcinomas will contain areas of glandular lumen. These areas should be ignored for scoring (Supplementary fig. 1).

### Smooth muscle tissue of the bowel wall

Smooth muscle tissue should be left out of the microscopic field (Fig. 2d). If this is not possible, the smooth muscle cells will have to be visually ignored for scoring (Table 2).

In T2-, T3-, and T4-staged adenocarcinomas of the colon, the tumor cells invade into or through the muscular layer of the colon. This can cause a mix-up of stromal cells and smooth muscle cells, which in some cases can be very hard to distinguish from one another. To enable an accurate scoring, we recommend performing an immunohistochemical desmin stain for these particular cases (Supplementary fig. 2).

### Blood vessels

Blood vessels are part of the stroma, and small vessels should therefore be included in the scoring, being a part of the neo-angiogenesis in the tumor micro-environment. However, fields of vision with native, large blood vessel(s) (i.e., thick smooth muscle wall of more than 3 layers of smooth muscle cells) should be replaced by another area for scoring, or, if this is not possible, the large vessel(s) should be visually ignored in the scoring (Supplementary Fig. 3a).

### Hyalinization

Hyalinization is a change in consistency of the collagenous matrix in the stromal tumor tissue, which gives the tissue a “glassy” appearance. Being a part of the stroma, it should be included in the scoring (Supplementary Fig. 3b).

### Tumor budding

Tumor budding occurs very often at the invasive front of adenocarcinomas of the colon [25]. Therefore, it is likely that cell clusters are located in a field of vision chosen for scoring the TSR. These very small cell clusters can sometimes be hard to distinguish in H&E stained sections, and they may, falsely, be ignored as adenocarcinoma cells in the TSR scoring. In those particular cases, when the (suspected) presence of budding cells makes it difficult to categorize the TSR estimate as low or high, it is recommended to perform an immunohistochemical cytokeratin stain (e.g., AE1/AE3) to identify these malignant epithelial tumor cells (Supplementary Fig. 4).

## Discussion

The high interest for the TSR, with sometimes differently used approaches of the protocol, calls for a standardized and easily implemented protocol. Although the technique described in this paper is focused on colon cancer, multiple studies have proven its robustness and usefulness for other types of solid epithelial cancers (Table 1). Our method and suggested protocol can therefore also be applied to these tumors. This also includes non-neoadjuvantly treated rectum carcinomas, as Park et al. showed in their study [14].

Scoring the TSR is a robust method, which only takes little extra time and costs, and has potential to be implemented in daily practice. The method is highly reproducible with low inter-observer variation (see Table 1). Nevertheless, some difficulties may appear during scoring, as discussed in this paper. In our experience, the biggest challenge is to distinguish between stromal tissue and smooth muscle fibers, particularly in stage II colon adenocarcinomas. In challenging cases, we recommend performing a desmin stain. Being an intermediate filament, desmin is expressed in both smooth and skeletal muscle myocytes. Although scoring the TSR is in general an easy to apply method, in any case of difficulties in scoring, or doubt by the observer, one may consult a second observer to his/her own need, according to the usual practice encountering challenging morphologies.

Also, in case of a stroma percentage at or around the cut-off point of 50%, consulting a second observer could be of help when in doubt. In addition, the total composition of the whole tissue section viewed with a × 5 objective could be considered to make a final decision.

Scoring of the TSR in colon adenocarcinomas is performed on the tissue slide from the most invasive part of the tumor, which is the slide used in routine pathology to determine the T status. This was decided after a study of colon cancers in which multiple H&E slides from different areas of the tumor were available for scoring. Although heterogeneity was seen in the percentage of stroma throughout the tumor, the highest stroma percentages were seen in the tumor areas with the deepest penetration in the bowel wall (higher T-stage) [4].

Most studies have validated our findings of the prognostic impact of the TSR in various kinds of malignant epithelial tumors. However, three studies have not been able to demonstrate validation of the TSR [22, 26, 27]. Discrepancies were caused by a different interpretation of the TSR scoring method. Instead of using the highest stroma percentage, these studies used either the mean percentage in case of heterogeneity [26], only one area of 9 mm^2^ at the tumor leading or non-leading edge [22], or the mean percentage of five image fields from not only the deepest invasive margin but also adjacent tumor areas [27]. The latter two studies both used semi-automated image analysis.

### Experimental design

Automated digitized estimation of the TSR allows for a broader and highly standardized application, and two international groups have actually validated our results using automated image analysis systems [17, 28]. Although this approach might increase reproducibility, such equipment is rather costly, and not accessible at all pathological departments yet. In addition, scanning and analyzing using an automated image analysis system takes approximately 20 min per slide. In contrast, visual microscopic scoring of the intra-tumor-stroma ratio can easily be performed as a routine for conventional morphological diagnosis, and therefore only takes a little extra time (< 2 min). Moreover, validation studies have independently reported an inter-observer reproducibility of substantial to almost perfect between two independent observers (Table 1). However, in the scope of digitizing the pathology workflow, automated scoring of the TSR would suit the diagnostic approach.

### Limitations

Assessment of the TSR can be adequately estimated in patients operated for a primary epithelial malignant neoplasm. Neo-adjuvant treatments with chemo- and/or radiotherapy induce changes to the cellular morphology and composition of the tumor microenvironment, and result in stromal formation surrounding the tumor [29–32]. Therefore, patients pre-treated with chemo- and/or radiotherapy should be excluded for TSR analysis. For these patients, analyzing pre-treatment biopsies might be a good alternative, although the TSR cannot be determined at the most invasive front. As biopsies for colon cancer are rare, this might not apply for these cases. However, the method described in this manuscript can be used for several other epithelial cancer types, for which taking biopsies is more common practice. This has been nicely demonstrated for example for esophageal cancer, with the TSR-scores of the tumor resection correlating with the matching pre-surgical biopsy TSR-scores in 81% of the cases studied. In discrepant cases, the biopsy scores were stroma-low, whereas the surgical removed tumors were scored stroma-high, thereby underestimating the TSR. For stroma-high cases, however, a 100% correlation was found. Moreover, TSR biopsy scores showed to be an independent prognostic factor for survival [33], which motivates more investigation into the prognostic and predictive impact of TSR in pre-treatment biopsies from malignant epithelial tumors.

## Electronic supplementary material

Supplementary fig. 1 (PNG 4520 kb)

High resolution image (TIF 12052 kb)

Supplementary fig. 2 (PNG 6411 kb)

High resolution image (TIF 20473 kb)

Supplementary fig. 3 (PNG 3409 kb)

High resolution image (TIF 10175 kb)

Supplementary fig. 4 (PNG 4369 kb)

High resolution image (TIF 14440 kb)
