# Supplementary figures and images for: Scoring the tumor-stroma ratio in colon cancer: procedure and recommendations
Source: Virchows Arch. 2018 Jul 20;473(4):405–12. doi: 10.1007/s00428-018-2408-z (PMC6182321; doi:10.1007/s00428-018-2408-z)

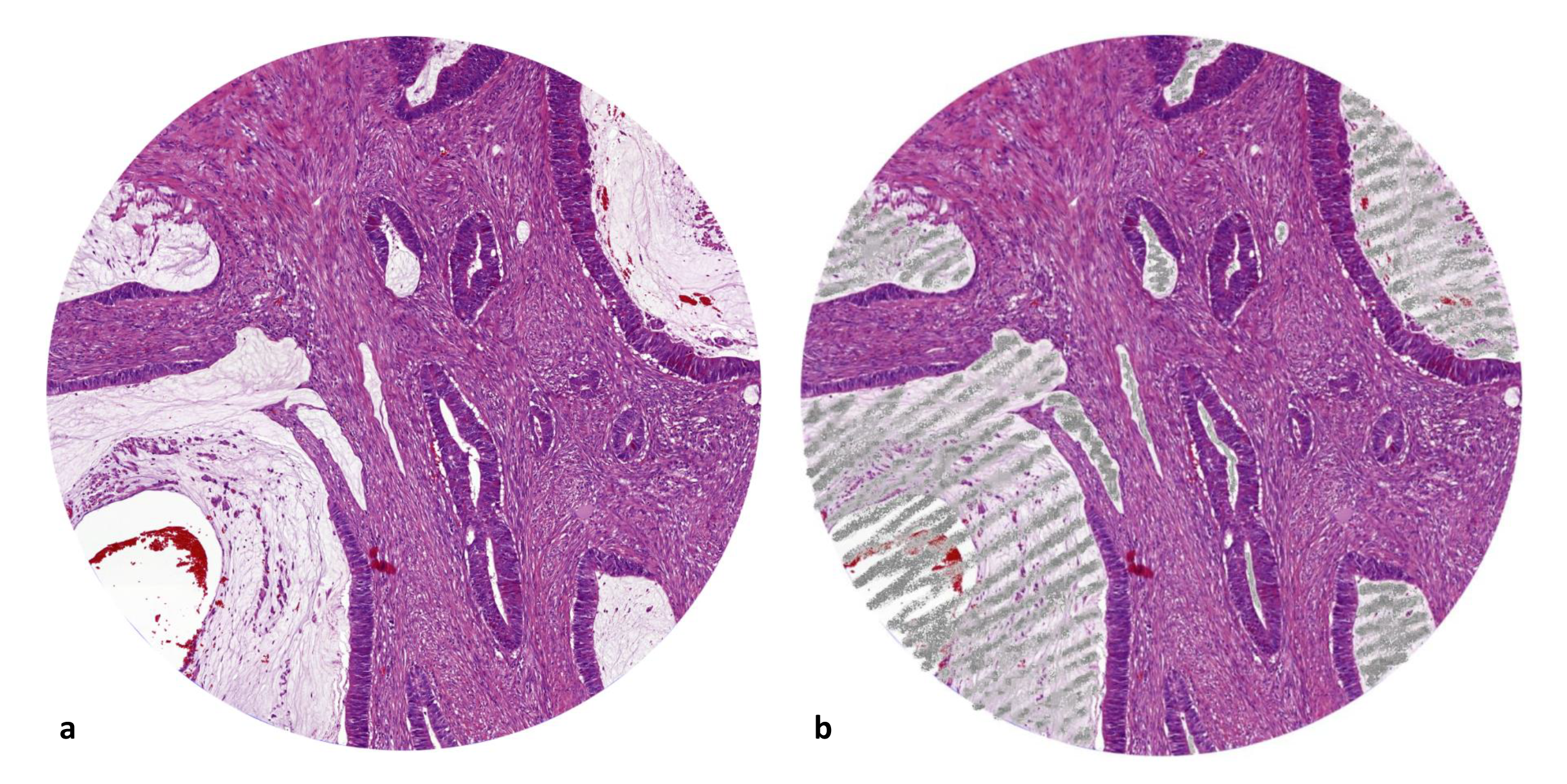

Supplement: Supplementary file 1 — (PNG 4520 kb) [file 428_2018_2408_Fig3_ESM.png]

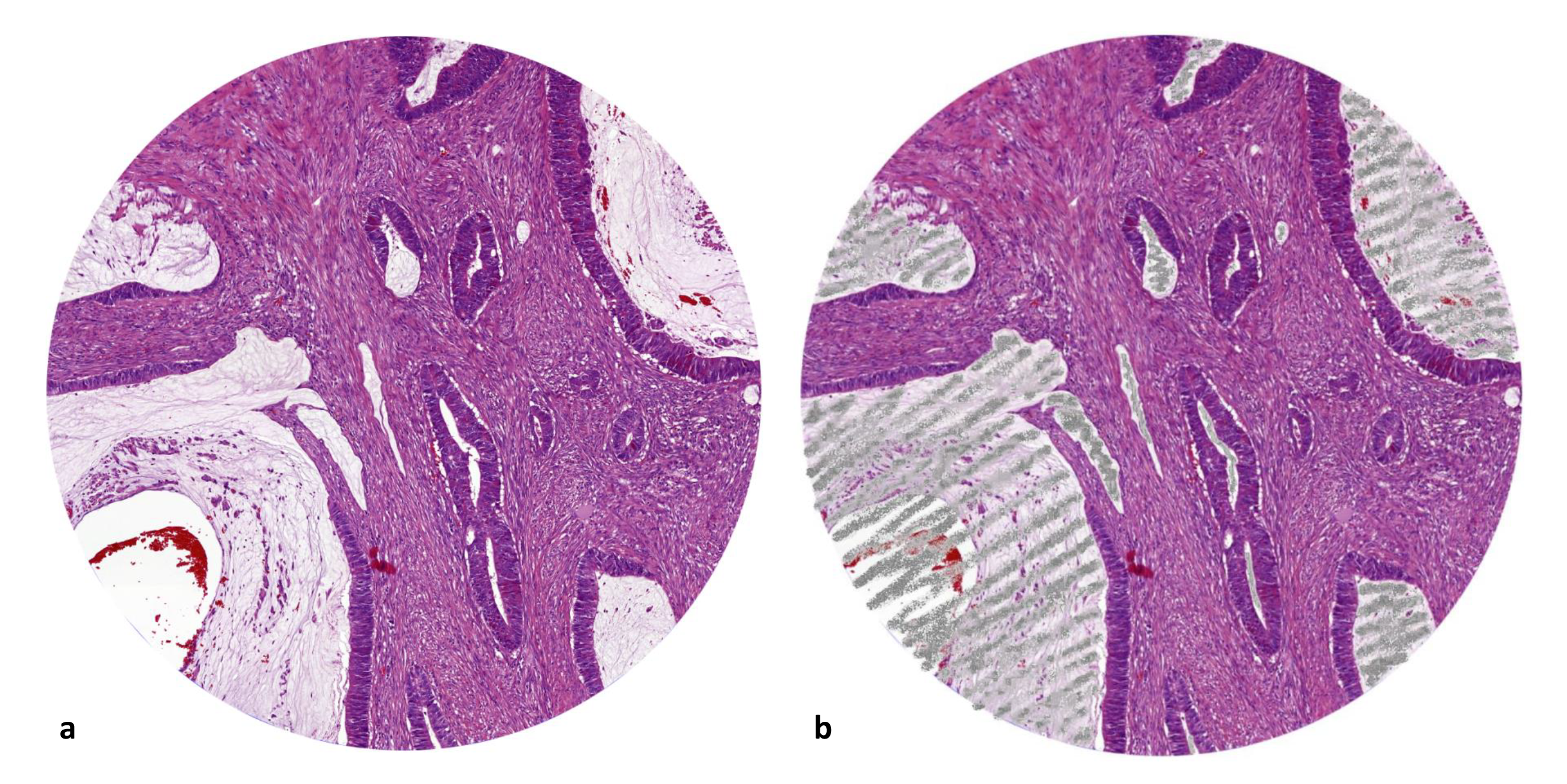

Supplement: Supplementary file 2 — High resolution image (TIF 12052 kb) [file 428_2018_2408_MOESM1_ESM.tif]

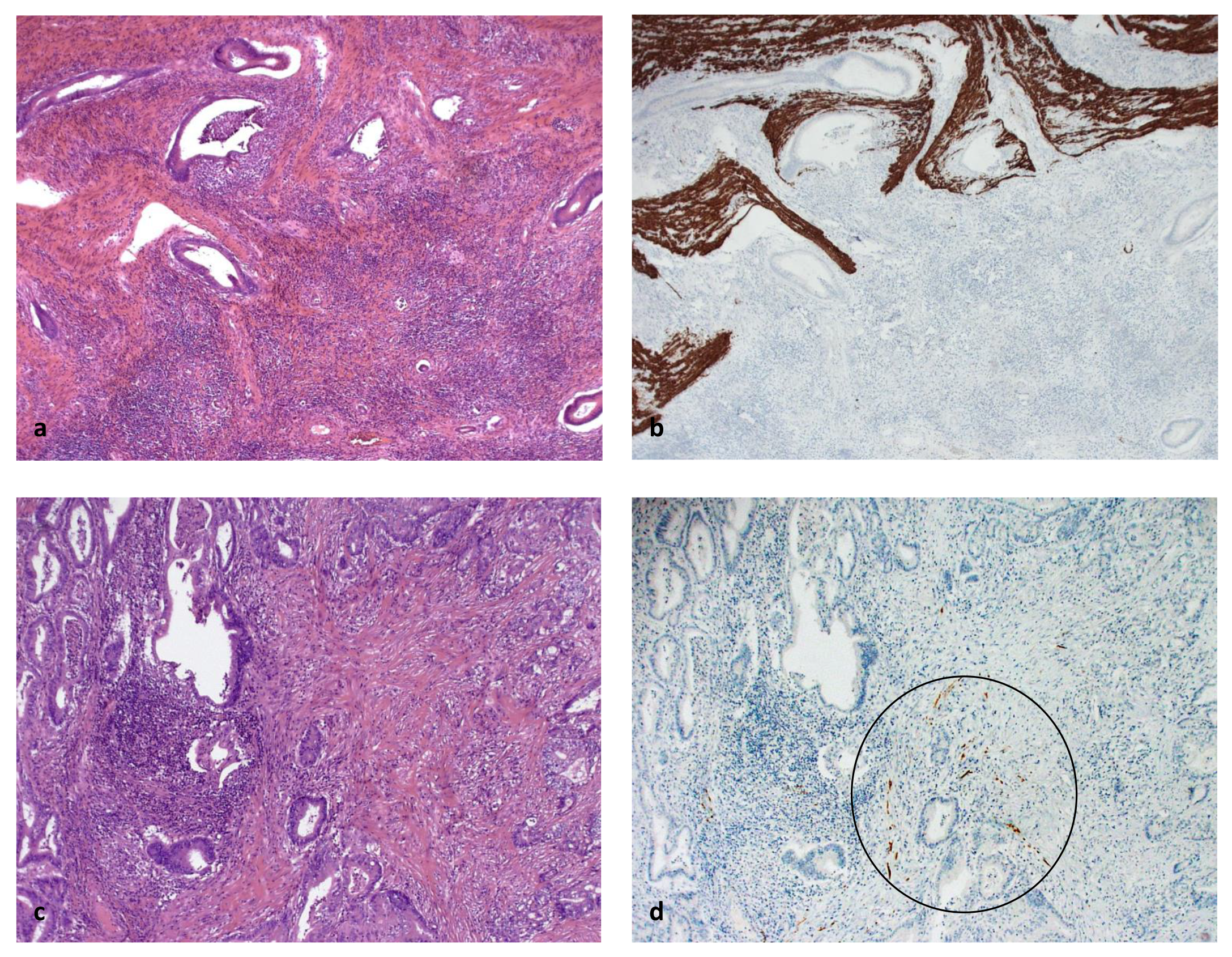

Supplement: Supplementary file 3 — (PNG 6411 kb) [file 428_2018_2408_Fig4_ESM.png]

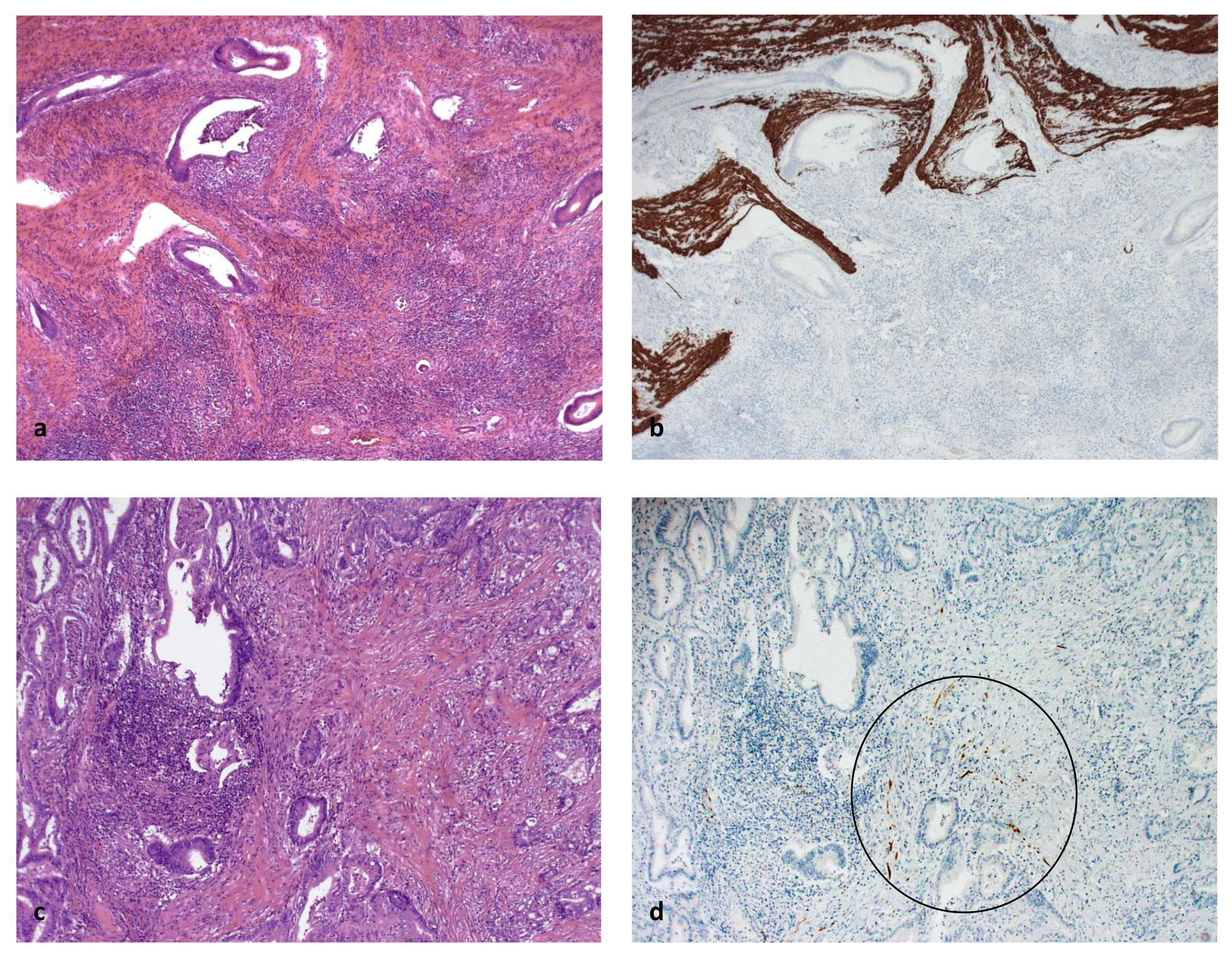

Supplement: Supplementary file 4 — High resolution image (TIF 20473 kb) [file 428_2018_2408_MOESM2_ESM.tif]

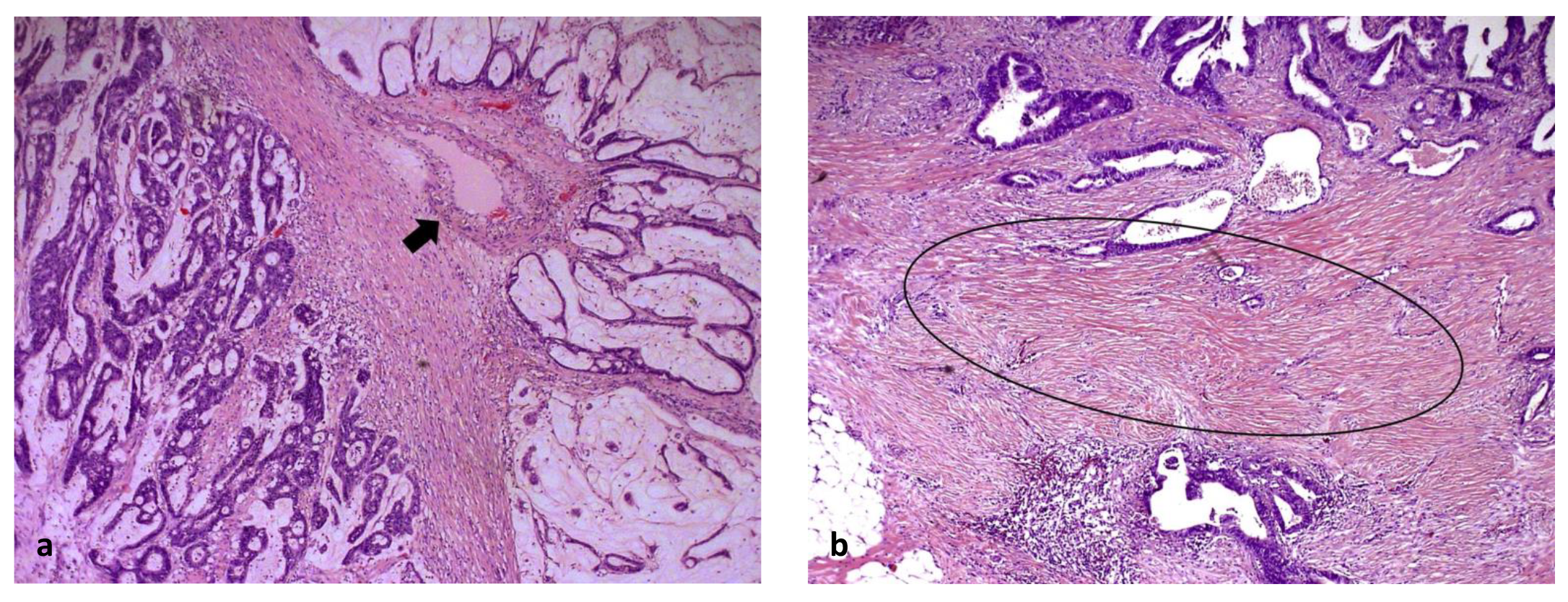

Supplement: Supplementary file 5 — (PNG 3409 kb) [file 428_2018_2408_Fig5_ESM.png]

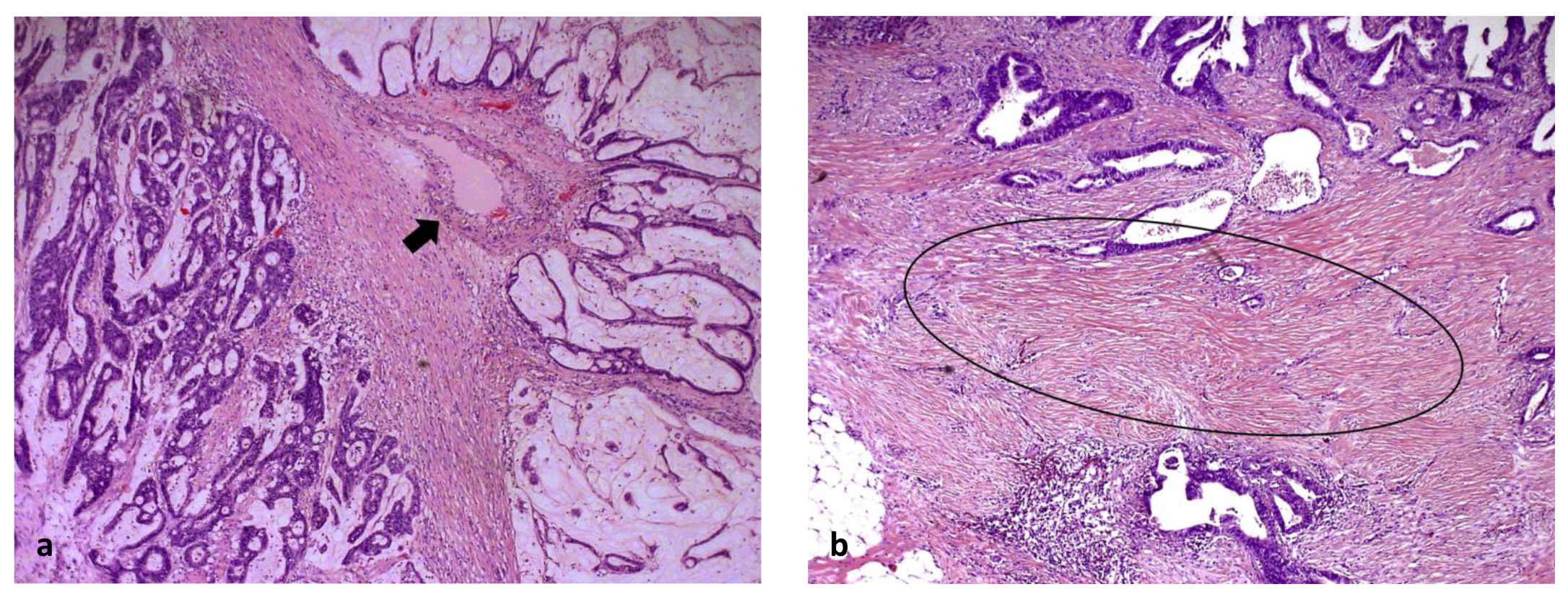

Supplement: Supplementary file 6 — High resolution image (TIF 10175 kb) [file 428_2018_2408_MOESM3_ESM.tif]

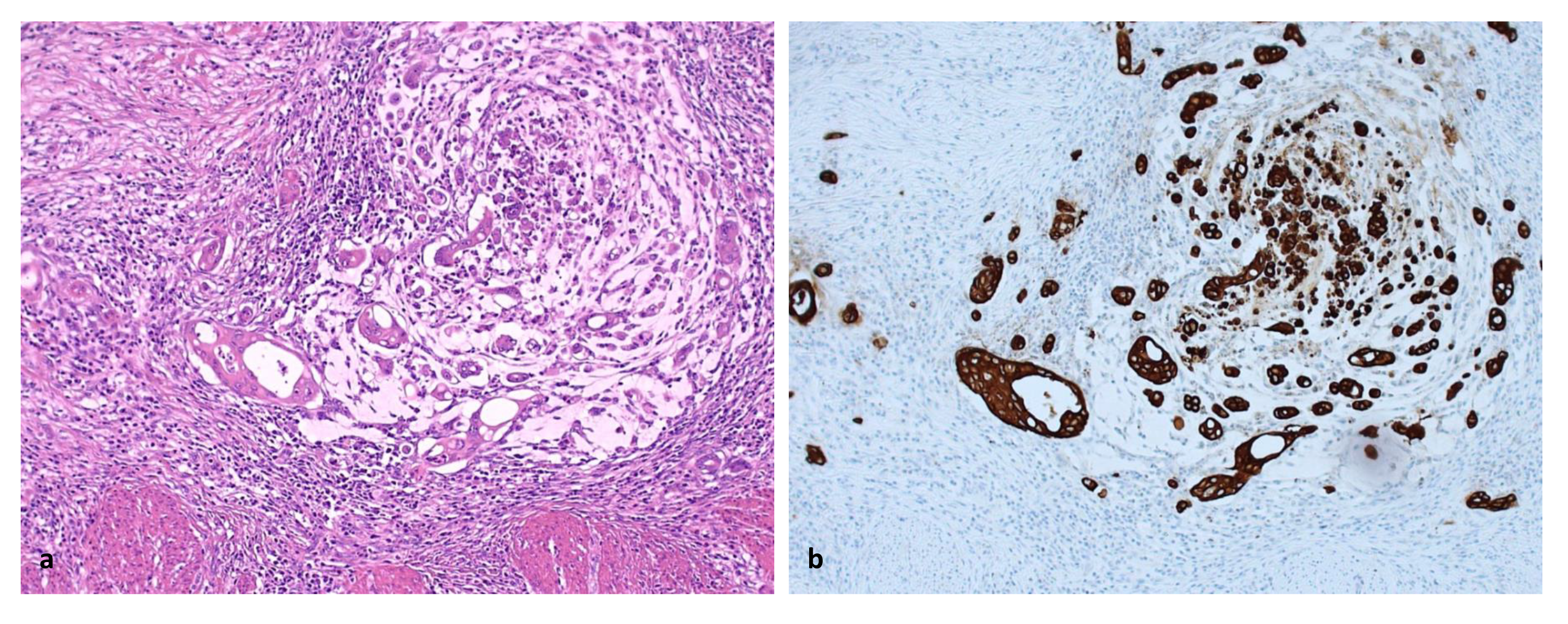

Supplement: Supplementary file 7 — (PNG 4369 kb) [file 428_2018_2408_Fig6_ESM.png]

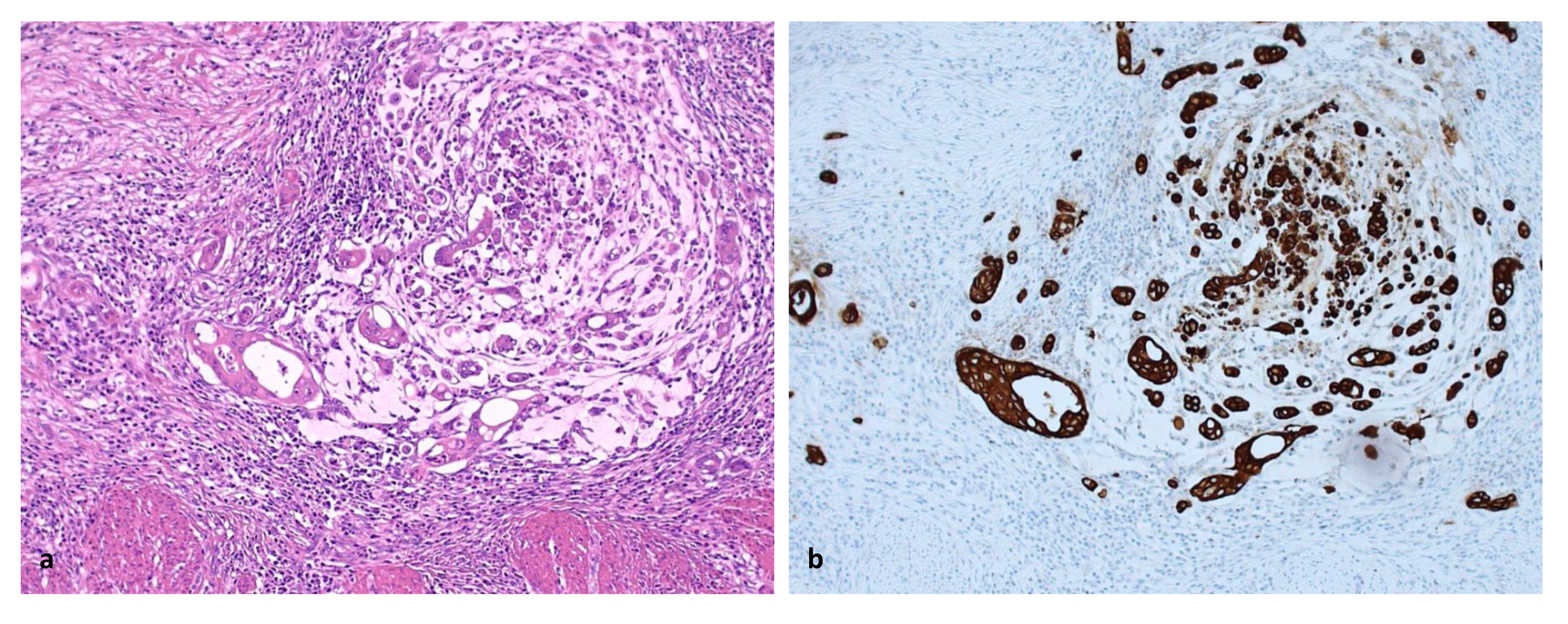

Supplement: Supplementary file 8 — High resolution image (TIF 14440 kb) [file 428_2018_2408_MOESM4_ESM.tif]
